# Low-cost outpatient chemotherapy regimen of cisplatin, 5-fluorouracil and leucovorin for advanced head and neck and esophageal carcinomas

**DOI:** 10.1590/S1516-31802008000100012

**Published:** 2008-01-03

**Authors:** Vanessa de Carvalho Fabrício, Fernanda Amado, Auro Del Giglio

**Keywords:** Head and neck neoplasms, Esophageal neoplasms, Antineoplastic combined chemotherapy protocols, Quality of life, Karnofsky performance status, Neoplasias de cabeça e pescoço, Neoplasias esofágicas, Protocolos de quimioterapia combinada antineoplásica, Qualidade de vida, Avaliação de estado de Karnofsky

## Abstract

**CONTEXT AND OBJECTIVE:**

Patients with advanced head and neck (H/N) and esophageal squamous cell carcinoma (SCC) often have a poor performance status and a dire prognosis. Our aim was to evaluate the feasibility, activity and quality of life (QOL) of an outpatient chemotherapy regimen consisting of cisplatin, 5-fluorouracil and leucovorin (CFL).

**DESIGN AND SETTING:**

Prospective phase II study conducted at a Brazilian public institution.

**METHODS:**

Fifteen patients with residual, recurrent or metastatic SCC of the H/N or esophagus received bolus infusions of leucovorin 20 mg/m^2^/day and 5-fluorouracil 370 mg/m^2^/day on days 1-4, and 90 minutes of infusion of cisplatin 25 mg/m^2^/day on days 1-3, every 21 to 28 days, depending on hematological recovery. We also evaluated QOL by applying the European Organization for Research and Treatment of Cancer Quality of Life-C30 questionnaire (EORTC QLQ-C30) before each cycle.

**RESULTS:**

The overall response rate was 36%, and the mean overall survival and progression-free survival were six and three months, respectively. We observed grade 3 or higher hematological toxicity in seven patients and one patient had grade 3 nausea and vomiting. One patient died because of neutropenic fever. Seven out of the 12 patients who could be evaluated regarding QOL presented an improvement in their overall health status and functional QOL scores over the course of the treatment.

**CONCLUSIONS:**

CFL is an active outpatient protocol with tolerable toxicity and a favorable QOL impact. Larger studies are warranted, in order to confirm these results.

**CLINICAL TRIAL REGISTRATION NUMBER:**

ISRCTN09659857

## RESUMO

**CONTEXTO E OBJETIVO:**

Pacientes com carcinoma espinocelular (CEC) de cabeça e pescoço e esôfago frequentemente tem um baixo Karnofsky Performance Status (KPS) e um prognóstico ruim. Nosso objetivo foi avaliar eficácia, taxa de resposta e qualidade de vida nesse contexto, em pacientes tratados com o regime ambulatorial cisplatina, 5-fluoruracil e leucovorin (CFL).

**TIPO DE ESTUDO:**

Estudo prospectivo fase II conduzido em uma instituição pública brasileira.

**MÉTODOS:**

15 pacientes com CEC de cabeça e pescoço e esôfago persistente, recorrente ou metastático receberam leucovorin 20 mg/m^2^/dia, *in bolus* seguido de 5-fluoruracil 370 mg/m^2^/dia, *in bolus* nos dias 1 a 4 e cisplatina 25 mg/m^2^/dia em infusão de 90 minutos nos dias 1 a 3 a cada 21 ou 28 dias, dependendo da recuperação hematológica. Qualidade de vida foi analisada utilizando o questionário EORTC QLQ-C30, aplicado antes de cada ciclo.

**RESULTADOS:**

A taxa de resposta objetiva foi de 36% e a sobrevida global e a sobrevida livre de progressão médias foram de 6,7 e 3,7 meses, respectivamente. Toxicidade hematológica maior ou igual a grau 3 foi observada em sete pacientes e um paciente apresentou náusea e vômito grau 3. Um paciente foi a óbito por neutropenia febril. Sete de 12 pacientes avaliáveis apresentaram melhora no estado geral de saúde e oito nas escalas funcional e sintomática com o tratamento.

**CONCLUSÃO:**

CFL é um protocolo ambulatorial factível, com toxicidade aceitável e com impacto favorável na qualidade de vida. Estudos maiores devem ser realizados para confirmar estes resultados.

## INTRODUCTION

Squamous cell carcinoma (SCC) of the head and neck and esophagus is a common type of neoplasia in developing countries such as Brazil. Chemotherapy is an important part of the multidisciplinary treatment for SCC patients, and the overall and clinical complete response rates for combined chemotherapy schemes among previously treated SCC patients are 15% and 55%, respectively.^1^ The most frequently used chemotherapy regimen includes four to five days continuous infusion of 5-fluorouracil and bolus cisplatin.^2-5^

In our country, the expense of pumps for continuous outpatient chemotherapy infusion, together with the scarcity of beds for elective admissions for palliative chemotherapy in an inpatient setting, prompted us to develop a protocol that could be administered without the need for hospital admission.

## OBJECTIVE

The present study had the aim of evaluating the combination of bolus cisplatin, 5-fluorouracil and leucovorin (CFL) in patients with advanced (residual, metastatic or recurrent) SCC of the head and neck and esophagus, with a view to obtaining a feasible and low-cost chemotherapy regimen to circumvent the need for infusion pumps and/or hospital admission.

## METHODS

Nonconsecutive patients with advanced or recurrent histologically confirmed head and neck and esophageal SCC were prospectively enrolled in this trial from January 2005 to June 2006, in Hospital Estadual Mário Covas, Santo André, São Paulo, Brazil. Since this was a pilot study, no sample size estimation was carried out before starting. All patients had to be 18 years of age or older, with normal renal function, measurable disease according to the response evaluation criteria in solid tumors (RECIST)^6^ and Karnofsky^7^ performance status (KPS) greater than or equal to 50%. This study had previously been approved by our institution's Research Ethics Committee.

After disease staging by means of computed tomography (CT) scans, the patients received leucovorin 20 mg/m^2^/day bolus infusions for four days (D1-D4), 5-fluorouracil 370 mg/m^2^/day bolus infusions for four days (D1-D4), and cisplatin 25 mg/m^2^/day in 90-minute infusions for three days (D1-D3), every 21 to 28 days, depending upon hematological recovery. This regimen was administered until hematological recovery or a state of intolerable toxicity was reached, or consent was withdrawn.

Quality of life (QOL) was evaluated using the European Organization for Research and Treatment of Cancer Quality of Life-C30 questionnaire (EORTC QLQ–C30)^8^ at the beginning of the study and before each cycle. This questionnaire had previously been used in Portuguese.^9,10^ Toxicity was analyzed in accordance with the National Cancer Institute (NCI) criteria^11^ before each cycle. KPS and clinical and laboratory parameters were also evaluated before each cycle. CT scans were repeated after third and sixth cycles of chemotherapy, in order to evaluate the responses.

## RESULTS

Between January 2005 and June 2006, 15 patients were enrolled and received a total of 45 cycles. The median was three cycles per patient (ranging from one to six). The patients’ characteristics are listed in Tables 1 and 2. It is important to note that 80% of the enrolled patients’ previous treatments had failed and their median KPS was 60%.

**Table 1.** Baseline patient characteristics

Variables	n (%)
**Age, years**	
Mean	62
Range	46-83
**Sex**	
Male	12 (80%)
Female	3 (20%)
**Education**
Elementary	5 (38.5%)
Middle grade	6 (46.2%)
High School	2 (15.4%)
**Marital Status**
Single	3 (20%)
Married	10 (66.7%)
Widow	2 (13.3%)
**Employed**	
Yes	3 (23.1%)
No	10 (76.9%)
**Initial Karnofsky performance status (%)**
Median	58.5
Range	43–91

(n = 15).

**Table 2.** Baseline tumor characteristics (n = 15)

Variables	n (%)
**Primary Site**	
Esophagus	4 (26.7%)
Pharynx	9 (60%)
Larynx	2 (13.3%)
**Recurrence**	
Local	6 (40%)
Distant	4 (26.7%)
Local and distant	2 (13.3%)
Metastatic at diagnosis	3 (20%)
**Initial treatment**	
None	3 (20%)
Surgery	1 (6.7%)
Surgery + radiotherapy	4 (26.7%)
Radiotherapy	1 (6.7%)
Radiotherapy + chemotherapy	5 (33.3%)
Surgery + radiotherapy + chemotherapy	1 (6.7%)

**Figure 1.**
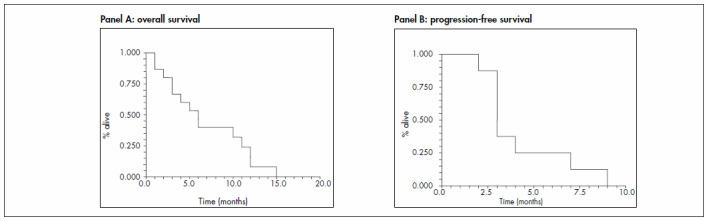
Kaplan-Meyer curves for overall survival (panel A) and progression-free survival (panel B) observed in our study.

Eleven patients could be evaluated with regard to response to treatment. Four patients were excluded from this analysis because they received fewer than three cycles of CFL (exclusion reasons: one with poor KPS, one death due to neutropenic fever and two lost from follow-up). The overall response rate was 36% (four partial responses) (95% CI: 7 to 65%). Four patients had stable disease, yielding a clinical benefit rate of 72% (95% CI: 45 to 99%). The mean progression-free survival was three months, and the overall survival for all 15 participants was six months (Figure 1).

The main observed toxicity was hematological: seven patients (53.9%) presented neutropenia of grade 3 or higher, two had neutropenic fever and one died as a consequence of it. Regarding nonhematological toxic effects, one patient had grade 3 nausea and vomiting.

**Figure 2.**
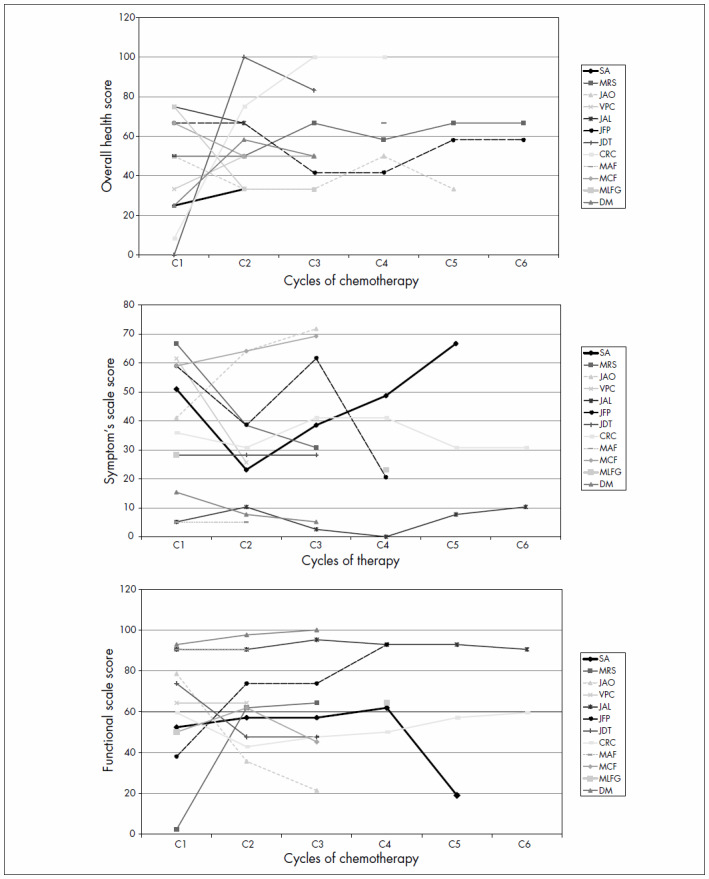
Patients’ quality of life (QOL) scores during the study according to the EORTC QLQ-C30 (European Organization for Research and Treatment of Cancer Quality of Life-C30). Overall health score (panel A), symptom scale (panel B) and functional scale (panel C).

Analysis of QOL using EORTC QLQ-C30 was only possible for 12 patients. Three patients received only one cycle and did not return afterwards. Seven patients (58%) presented an improvement in their overall health status and functional QOL scores over the course of the treatment (Figure 2). We found no correlations between response to chemotherapy and improvement in QOL.

## DISCUSSION

Treatments for head and neck and esophageal SCC have evolved over recent years through the introduction of modern irradiation techniques integrated with chemotherapy. Notwithstanding these advances, most patients still present with advanced disease and many of them end up progressing and eventually dying because of their disease.^12-15^ Therefore, it has become important to develop feasible, cheap and effective chemotherapy protocols to palliate symptoms and improve QOL.

The overall response rate with single-agent chemotherapy for patients with recurrent or metastatic head and neck cancer ranges from 15 to 30%, depending on performance status, previous treatments and tumor burden.^16^ With the use of polychemotherapy, the response rates increase to 20 to 35%, although without significant improvement in survival.^16,17^ In fact, the median survival in studies that included infused 5-fluorouracil and/or taxanes ranged from five to nine months.^16,17^ In our study, we found a response rate of 36% and median overall survival of six months, in a population of patients who had mostly had previous treatment and presented poor performance and high tumor burden.

The main toxic effect that we observed with CFL was myelotoxicity, which was in agreement with previous reports of grade 3 or higher hematological toxicity at rates of 33%^18^ to 65%,^19^ in regimens containing continuous infusion of 5-fluorouracil and cisplatin. Moreover, despite this toxicity, most of our CFL-treated patients who could be evaluated experienced QOL improvements in relation to their overall health and symptom scale scores.

## CONCLUSION

CFL seems to be a feasible outpatient protocol for advanced and recurrent head and neck and esophageal SCC, with tolerable toxicity and a favorable QOL impact. Further studies are warranted, in order to test CFL on larger numbers of patients.

## References

[1] Gebbia V, Testa A, Valenza R (1993). A phase II study of levofolinic acid and 5-fluorouracil plus cisplatin in patients with advanced head and neck squamous cell carcinoma. Oncology.

[2] Lee JJ, Jeng JH, Wang HM (2005). Univariate and multivariate analysis of prognostic significance of betel quid chewing in squamous cell carcinoma of buccal mucosa in Taiwan. J Surg Oncol.

[3] Pignon JP, Bourhis J, Domenge C, Designé L (2000). Chemotherapy added to locoregional treatment for head and neck squamous-cell carcinoma: three meta-analyses of updated individual data. MACH-NC Collaborative Group. Meta-Analysis of Chemotherapy on Head and Neck Cancer. Lancet.

[4] Clavel M, Vermorken JB, Cognetti F (1994). Randomized comparison of cisplatin, methotrexate, bleomycin and vincristine (CABO) versus cisplatin and 5-fluorouracil (CF) versus cisplatin (C) in recurrent or metastatic squamous cell carcinoma of the head and neck. A phase III study of the EORTC Head and Neck Cancer Cooperative Group. Ann Oncol.

[5] Colevas AD (2006). Chemotherapy options for patients with metastatic or recurrent squamous cell carcinoma of the head and neck. J Clin Oncol.

[6] Therasse P, Arbuck SG, Eisenhauer EA (2000). New guidelines to evaluate the response to treatment in solid tumors. European Organization for Research and Treatment of Cancer, National Cancer Institute of the United States, National Cancer Institute of Canada. J Natl Cancer Inst.

[7] Conill C, Verger E, Salamero M (1990). Performance status assessment in cancer patients. Cancer.

[8] Ringdal GI, Ringdal K (1993). Testing the EORTC Quality of Life Questionnaire on cancer patients with heterogeneous diagnoses. Qual Life Res.

[9] Souza RCC, Barros CAA, Souza RRL (2005). Avaliação da qualidade de vida de doentes de carcinoma retal, submetidos à ressecção com preservação esfincteriana ou à amputação abdômino-perineal. [Evaluation of life quality of sick people of rectal carcinoma, submitted to the resection with sphincter preservation or the abdominoperineal amputation]. Rev Bras Colo-proctol.

[10] Samano ES, Goldenstein PT, Ribeiro Lde M (2004). Praying correlates with higher quality of life: results from a survey on complementary/alternative medicine use among a group of Brazilian cancer patients. Sao Paulo Med J.

[11] National Cancer Institute CTC v2.0 and Common Terminology Criteria for Adverse Events v3.0 (CTCAE).

[12] Olmi P, Crispino S, Fallai C (2003). Locoregionally advanced carcinoma of the oropharynx: conventional radiotherapy vs. accelerated hyperfractionated radiotherapy vs. concomitant radiotherapy and chemotherapy--a multicenter randomized trial. Int J Radiat Oncol Biol Phys.

[13] Denis F, Garaud P, Bardet E (2004). Final results of the 94-01 French Head and Neck Oncology and Radiotherapy Group randomized trial comparing radiotherapy alone with concomitant radiochemotherapy in advanced-stage oropharynx carcinoma. J Clin Oncol.

[14] Ang KK, Harris J, Garden AS (2005). Concomitant boost radiation plus concurrent cisplatin for advanced head and neck carcinomas: radiation therapy oncology group phase II trial 99-14. J Clin Oncol.

[15] Zorat PL, Paccagnella A, Cavaniglia G (2004). Randomized phase III trial of neoadjuvant chemotherapy in head and neck cancer: 10-year follow-up. J Natl Cancer Inst.

[16] Forastiere A, Koch W, Trotti A, Sidransky D (2001). Head and neck cancer. N Engl J Med.

[17] Wong SJ, Machtay M, Li Y (2006). Locally recurrent, previously irradiated head and neck cancer: concurrent re-irradiation and chemotherapy, or chemotherapy alone?. J Clin Oncol.

[18] Webb A, Cunningham D, Scarffe JH (1997). Randomized trial comparing epirubicin, cisplatin, and fluorouracil versus fluorouracil, doxorubicin, and methotrexate in advanced esophagogastric cancer. J Clin Oncol.

[19] Vokes EE, Schilsky RL, Weichselbaum RR (1989). Cisplatin, 5-fluorouracil, and high-dose oral leucovorin for advanced head and neck cancer. Cancer.

